# Maternal Methylenetetrahydrofolate Reductase C677T Polymorphism and Down Syndrome Risk: A Meta-Analysis from 34 Studies

**DOI:** 10.1371/journal.pone.0108552

**Published:** 2014-09-29

**Authors:** Vandana Rai, Upendra Yadav, Pradeep Kumar, Sushil Kumar Yadav, Om Prakesh Mishra

**Affiliations:** 1 Human Molecular Genetics Laboratory, Department of Biotechnology, VBS Purvanchal University, Jaunpur, India; 2 Department of Pediatrics, Institute of Medical Sciences, Banaras Hindu University, Varanasi, India; Sanjay Gandhi Medical Institute, India

## Abstract

**Background:**

Methylenetetrahydrofolate reductase (*MTHFR*) is a key enzyme of folate metabolic pathway which catalyzes the irreversible conversion of 5, 10-methylenetetrahydrofolate to 5-methyltetrahydrofolate. 5-methyltetrahydrofolate donates methyl group for the methylation of homocysteine to methionine. Several studies have investigated maternal MTHFR C677T polymorphism as a risk factor for DS, but the results were controversial and inconclusive. To come into a conclusive estimate, authors performed a meta-analysis.

**Aim:**

A meta-analysis of published case control studies was performed to investigate the association between maternal MTHFR C677T polymorphism and Down syndrome.

**Methods:**

PubMed, Google Scholar, Elsevier, Springer Link databases were searched to select the eligible case control studies using appropriate keywords. The pooled odds ratio (OR) with 95%confidence interval were calculated for risk assessment.

**Results:**

Thirty four studies with 3,098 DS case mothers and 4,852 control mothers were included in the present meta-analysis. The pooled OR was estimated under five genetic models and significant association was found between maternal *MTHFR* 677C>T polymorphism and Down syndrome under four genetic models except recessive model (for T vs. C, OR = 1.26, 95% CI = 1.09–1.46, p = 0.001; for TT vs. CC, OR = 1.49, 95% CI = 1.13–1.97, p = 0.008; for CT vs. CC, OR = 1.29, 95% CI = 1.10–1.51, p = 0.001; for TT+CT vs. CC, OR = 1.35, 95% CI = 1.13–1.60, p = 0.0008; for TT vs. CT+CC, OR = 0.76, 95% CI = 0.60–0.94, p = 0.01).

**Conclusion:**

The results of the present meta-analysis support that maternal *MTHFR* C677T polymorphism is a risk factor for DS- affected pregnancy.

## Introduction

Down syndrome (DS) is the most common chromosomal disorder with the prevalence of 1/700–1000 live birth. It is characterized by the trisomy 21, which results from maternal meiotic nondisjunction in majority (90%) of cases. The established risk factor for DS is advanced (>35 years) maternal age at the time of conception. However, a fairly high number of DS children born to younger mothers suggest that risk factors other than advanced maternal age might be involved in predisposing younger mothers to DS-affected pregnancy [1], [2]. The molecular and biochemical mechanism of maternal meiotic non-disjunction is still not known. James et al. [3] reported that methylenetetrahydrofolate reductase (*MTHFR*) C677T polymorphism might be a risk factor for maternal meiotic non-disjunction. Since then several studies have investigated the risk of DS to variants of folate pathway genes like *MTHFR*, Methionine synthase (*MTR*) and Methionine synthase reductase (*MTRR*) in Asian [1], [2], [4], [5] and Caucasian [6]–[8] populations. Folate deficiency and dysfunctional MTHFR causes abnormal DNA methylation [9], [10] and chromosomal segregation [11], [12]. Hypomethylation of the centromeric DNA has been suggested as the causative mechanism of meiotic non-disjunction. Abnormal DNA methylation of centromere lead to aberrant kinetochore formation that results into abnormal segregation of chromosomes during meiosis [3], [13].

MTHFR is a key enzyme in folate metabolism, which catalyzes the reduction of 5, 10-methylenetetrahydrofolate to the predominant circulating form of folate i.e. 5-methyltetrahydrofolate (5-THF). 5-THF donates methyl group for the conversion of homocysteine to methionine, which is further converted into S-adenosylmethionine (SAM). SAM is the main methyl group donor for all cellular methylation reactions. Folate deficiency and/or dysfunctional MTHFR reduces the conversion of 5, 10-methylene THF to 5-methyl THF, and elevates plasma homocysteine concentration. Both folate and MTHFR are involved in many complex biochemical reactions like DNA synthesis, repair and methylation.

There are more than 40 polymorphisms reported in *MTHFR* gene and among them C677T variant is the most studied and clinically important. The C677T variant (rs 1801133; Ala 222 Val) has been associated with a decreased activity of MTHFR, and increased homocysteine level [14]–[16]. Mutant homozygous (TT) individuals have a decreased enzymatic activity ∼ 70% and the heterozygote by 40%. A dysfunctional MTHFR leads to lower levels of SAM resulting into DNA hypomethylation. DNA hypomethylation increases the risk of many diseases and disorders like- neural tube defects [17], cleft lip and palate [18], Alzheimer disease [19], cardiovascular diseases [14], diabetes [20] and psychiatric disorders [21] etc. Several epidemiological studies have investigated the associations of the maternal *MTHFR* C677T polymorphism with Down syndrome. However, the results were conflicting and inconclusive. In light of the above facts, we conducted a meta-analysis of published case control studies relating the C677T polymorphism of the maternal *MTHFR* gene to the risk of having DS offspring.

## Materials and Methods

### Selection of studies

Electronic searches were conducted using PubMed, Google Scholar, Elsevier and Springer link and all published manuscripts up to January, 2014 were considered in present meta-analysis. The following index terms were used for search ‘*MTHFR*’ ‘Methylenetetrahydrofolate reductase’, and ‘C677T polymorphism’, ‘maternal risk’ and ‘Down syndrome’. In addition, bibliographies of all articles and reviews were hand searched for additional suitable studies.

### Inclusion criteria

Included studies had to meet the following criteria: (1) article should be published; (2) article should have sufficient data to calculate the odds ratio with 95% CI; (3) article should be case control association study; and (4) author should describe the genotyping protocols.

### Data extraction

The following data were extracted from each study: first author’s name, publication year, journal name, country name, genotyping method, and different *MTHFR* genotype numbers.

### Meta-analysis

Statistical analysis of maternal *MTHFR* C677T polymorphism and DS risk was estimated by Odds ratio (ORs) with 95% confidence intervals (CIs). The heterogeneity was tested by the Q-statistics with p-values <0.05. Subgroup analysis was done to know the source of heterogeneity. If higher heterogeneity (I^2^>50%) would be observed, the random effect model [22] would be applied. Otherwise, fixed-effect model [23] was applied to obtain the summary OR and 95% CI. All p values were two-sided and a p value of less than 0.05 was considered statistically significant. All analyses were performed using the computer program MIX version 1.7 [24]. The control genotypes were tested for Hardy-Weinberg equilibrium (HWE) using the Goodness of fit Chi-square test. The quality of the included studies was measured according to the scoring system for randomized controlled association studies proposed by Clark and Baudouin [25]. Case control studies scoring <5 were defined as low quality study and those ≥5 were defined as high quality study.

### Publication bias

Funnel plots of precision by log (OR) and standard error by log (OR) were plotted to determine publication bias and asymmetrical funnel plots represent publication bias. Begg and Mazumdar rank correlation [26] and Egger’s regression intercept [27] tests were adopted to assess the publication bias.

## Results

### Eligible Studies

**Figure 1:**
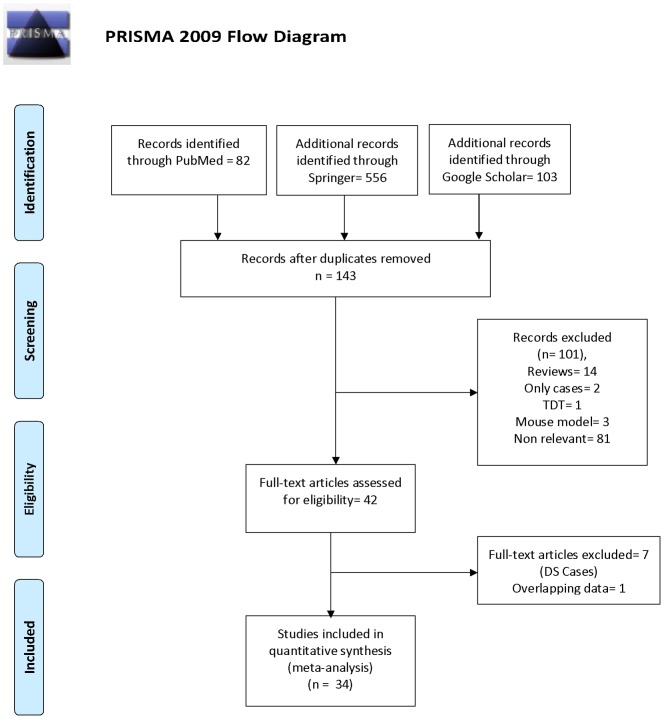
Flow Diagram of Study Searching and Selection Process.

With our original search criterion, 85 articles were found. After reviewing each original article, 50 publications were excluded including reviews, case studies, editorials etc. (Figure 1). Following these exclusions, 34 individual case-control studies with a total of 3,098 cases and 4,852 controls were found to be suitable for inclusion into meta-analysis and listed in Table 1 (Figure 1).

**Table 1:** Characteristics of the eligible studies included in the meta-analysis.

Study	Year	Country	Case	Control	Quality Score	Reference
James et al.	1999	Canada	50	57	7	Am J Clin Nutr 70∶495-50
Hobbs et al.	2000	America	157	140	7	Am J Hum Genet 67∶623–630
Chadeaux-Vekemans et al.	2002	France	85	70	5	Pediatr Res 51∶766–767
O’Leary et al.	2002	Ireland	41	192	5	Am J Med Genet A 107∶151–155
Stuppia et al.	2002	Italy	64	112	7	Eur J Hum Genet 10∶388–390
Boduroglu et al.	2004	Turkey	158	91	5	Am J Med Genet 127A: 5–10
Acacio et al.	2005	Brazil	70	88	8	Prenat Diagn 25∶1196–1199
Da Silva et al.	2005	Brazil	154	158	7	Am J Med Genet Part A 135A: 263–267
Coppede et al.	2006	Italey	79	111	7	Am J Med Genet A 140(10): 1083–1091
Liang et al.	2006	China	30	70	7	China J Modern Medicine 20∶011
Rai et al.	2006	India	149	165	6	J Hum Genet 51∶278–283
Scala et al.	2006	Italy	94	256	8	Genet Med 8∶409–416
Wang et al.	2007	China	100	100	8	Zhonghua Yi Xue Yi Chuan Xue Za Zhi 24∶533–537
Biselli et al.	2008	Brazil	82	134	8	Genet Mol Res 7∶33–42
Kohli et al.	2008	India	103	109	6	Downs Syndr Res Pract 12∶133–137
Martinez-Frias et al.	2008	Spain	146	188	4	Am J Med Genet A 146A(11): 1477–1482
Meguid et al.	2008	Egypt	42	48	7	Dis Markers 24∶19–26
Santos-Reboucas et al.	2008	Brazil	103	108	7	Dis Markers 25∶149–157
Wang et al.	2008	China	64	70	8	J Zhejiang Univ Sci B 9(2): 93–99
Brandalize et al.	2009	Brazil	239	197	6	Am J Med Genet 149A (10): 2080–2087
Coppede et al.	2009	Italy	94	113	8	Neurosci Lett 449∶15–19
Cyril et al.	2009	India	36	60	6	Indian J Hum Genet 15∶60–64
Kokotas et al.	2009	Denmark	177	984	6	Dis Markers 27∶279–285
Pozzi et al.	2009	Italy	74	184	8	Am J Obstet Gynecol 63: e1–e6
Coppede et al.	2010	Italy	29	32	5	BMC Med Genomics 3∶42
Liao et al.	2010	China	60	68	7	Yi Chuan 32(5): 461–466
Vranekoviz et al.	2010	Croatia	111	141	7	Dis Markers 28∶293–298
Bozovic et al.	2011	Croatia	112	221	7	Pediatr Int 53(4): 546–550
Sadiq et al.	2012	Jordan	53	29	6	Genet Test Mol Biomarker 15∶1–7
Tayeb	2012	Saudi Arabia	30	40	5	Egyptian J Med Hum Genet 13(3): 263–268
Zampieri et al.	2012	Brazil	105	185	8	Dis Markers 32(2): 73–81
Kaur and Kaur	2013	India	110	111	6	Indian J Hum Genet 19(4): 412–414
Pandey et al.	2013	India	81	99	6	Int J Pharm Bio Sci; 4(2):(B)249–256
Elsayed et al.	2014	Egypt	26	61	9	The Egyptian J Med Hum Genet 15(1): 39–44

These studies were published between 1999 and 2013. All these thirty four studies were performed in different countries- Brazil [28]–[33], China [4], [34]–[36], Croatia [8], [37], Egypt [38], [39], France [40], India [1], [5], [41]–[43], Ireland [44], Italy [7], [13], [45]–[48], Jordan [49], Netherlands [50], Saudi Arabia [2], Spain [51], Turkey [52] and USA [3], [6] (Table 1).

### Characteristics of included studies

In thirty four studies included in the present meta-analysis, the smallest case sample size was 26 [39] and highest sample size was 239 [32]. ORs for more than one were reported in twenty four articles [1], [2], [4]–[6], [8], [13], [28]–[30], [32], [33], [35]–[39], [42], [43], [46]–[49], [51], [52]. Except two studies [28], [43], control populations of all articles were in Hardy-Weinberg equilibrium.

In all thirty four studies, total cases were 3,098 with CC (1,396), CT (1,326) and TT (376), and controls were 4,852 with CC (2,329), CT (2,015), and TT (508) genotypes. In controls genotypes, percentage of CC, CT and TT were 48.00%, 41.53%, and 10.47% respectively. In total cases, genotype percentage of CC, CT, and TT was 45.06%, 42.8% and 12.14% respectively. Frequencies of CC and CT genotypes were highest in both cases and controls (Table 2). In cases and controls, the allele C was the most common. All five genetic models; -allele contrast (T vs C) homozygote (TT vs CC), codominant (CT vs CC), dominant (TT+CT vs CC) and recessive (TT vs CT+CC) models were used to evaluate C677T polymorphism as DS risk.

**Table 2:** Distributions of MTHFR C677T genotypes and allele frequencies in DS case mothers and control mothers reported in different included studies.

		CC		CT		TT		C		T	
Study	Country	Case	Control	Case	Control	Case	Control	Case	Control	Case	Control
James et al., 1999	Canada	24	15	22	34	4	8	70	64	30	50
Hobbs et al., 2000	America	51	67	84	59	22	14	186	193	128	87
Chadeaux-Vekemans et al., 2002	France	36	29	42	30	7	11	114	88	56	52
O’Leary et al., 2002	Ireland	18	90	21	84	2	18	57	264	25	120
Stuppia et al., 2002	Italy	20	27	32	62	12	23	72	116	56	108
Boduroglu et al., 2004	Turkey	86	58	55	30	17	3	227	146	89	36
Acacio et al., 2005	Brazil	35	54	30	25	5	9	100	133	40	43
Da Silva et al., 2005	Brazil	67	84	72	67	15	7	206	235	102	81
Coppede et al., 2006	Italey	20	39	43	54	16	18	83	132	75	90
Liang et al., 2006	China	7	16	20	34	3	20	34	66	26	74
Rai et al., 2006	India	97	124	40	39	12	2	234	287	64	43
Scala et al., 2006	Italy	31	74	39	125	24	57	101	273	87	239
Wang et al., 2007	China	28	48	52	42	20	10	108	138	92	62
Biselli et al., 2008	Brazil	29	100	35	77	8	17	93	229	71	39
Kohli et al., 2008	India	74	71	29	32	0	6	177	174	29	44
Martinez-Frias et al., 2008	Spain	61	76	61	85	24	27	183	237	109	139
Meguid et al., 2008	Egypt	20	33	17	12	5	3	57	78	27	18
Santos-Reboucas et al., 2008	Brazil	51	49	43	47	9	12	145	145	61	71
Wang et al., 2008	China	14	36	32	29	18	5	60	101	68	39
Brandalize et al., 2009	Brazil	94	86	113	93	32	18	301	265	177	129
Coppede et al., 2009	Italy	25	40	52	55	17	18	102	135	86	91
Cyril et al., 2009	India	33	60	3	0	0	0	69	120	3	0
Kokotas et al., 2009	Denmark	92	445	72	449	13	90	256	1339	98	629
Pozzi et al., 2009	Italy	28	62	30	93	16	29	86	217	62	151
Coppede et al., 2010	Italy	5	11	19	17	5	4	29	39	29	25
Liao et al., 2010	China	12	23	26	33	22	12	50	79	70	57
Vranekoviz et al., 2010	Croatia	49	66	49	64	13	11	147	196	75	86
Bozovic et al., 2011	Croatia	46	101	55	97	11	23	147	299	77	143
Sadiq et al., 2011	Jordan	23	23	27	5	3	1	73	51	33	7
Tayeb, 2012	Saudi Arabia	16	22	10	14	4	4	42	58	18	22
Zampieri et al., 2012	Brazil	40	94	55	73	10	18	135	261	75	109
Kaur & Kaur, 2013	India	86	89	22	22	2	0	194	200	26	22
Pandey et al., 2013	India	67	87	12	9	2	3	146	183	16	15
Elsayed et al., 2014	Egypt	11	30	12	24	3	7	34	84	18	38

### Meta-analysis

**Figure 2:**
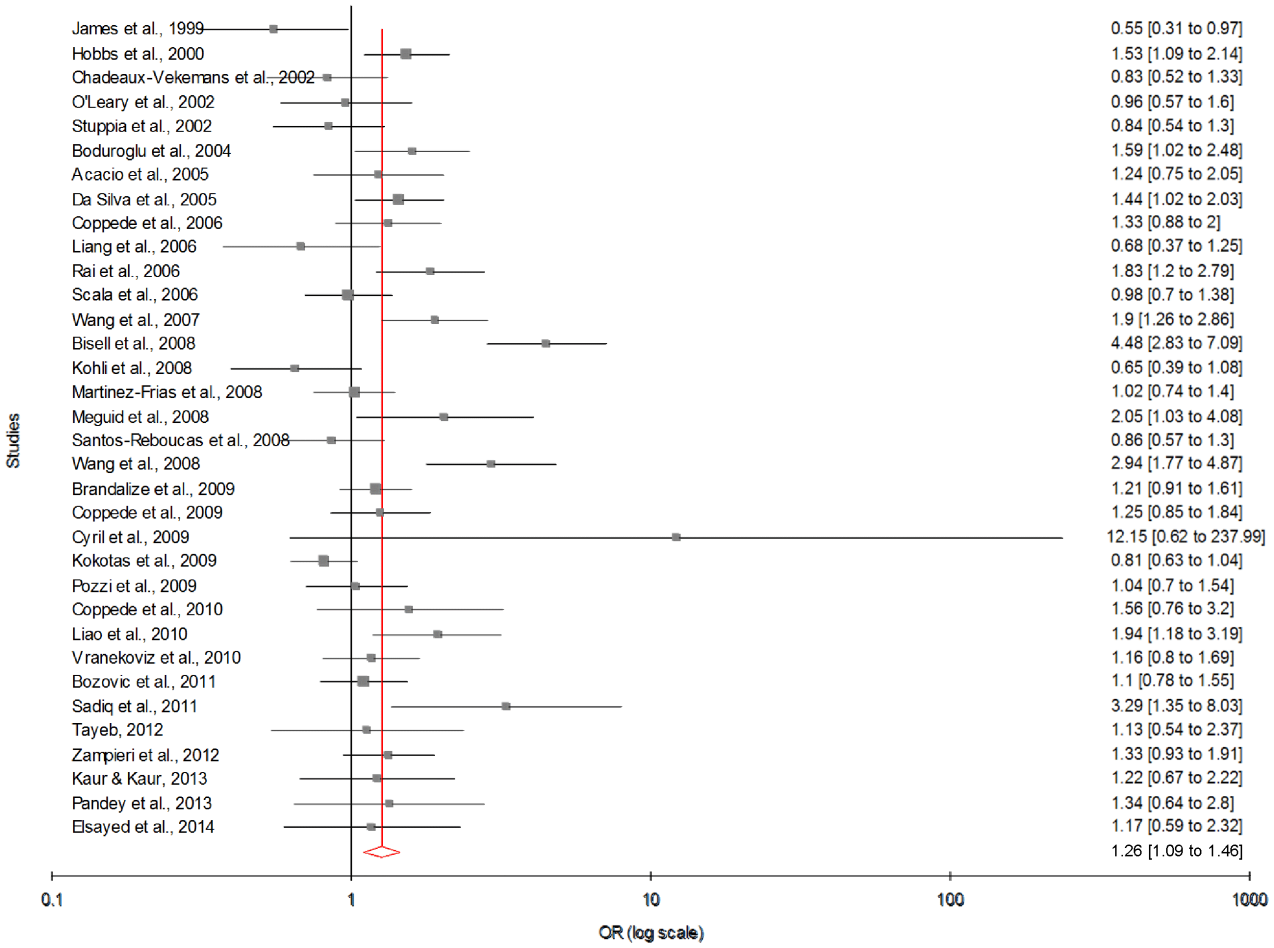
Forest plots (Random effect) showed significant association between MTHFR C677T polymorphism and risk of Down syndrome using allele contrast model (C versus T). Results of individual and summary OR estimates and 95% CI of each study were shown. Horizontal lines represented 95% CI, and dotted vertical lines represent the value of the summary OR.

Meta-analysis with allele contrast showed significant association between maternal 677T allele and DS with both fixed effect (OR_TvsC_ = 1.22; 95% CI = 1.13–1.31; p = <0.0001) and random effect models (OR_TvsC_ = 1.26; 95% CI = 1.09–1.45; p = 0.001) (Figure 2) (Table 3). In cumulative meta-analysis using random effect model, the association of maternal T allele with DS turned statistically significant with the addition of study of Wang et al. (2008) and remained significant thereafter.

**Table 3:** Summary estimates for the odds ratio (OR) of MTHFR C677T in various allele/genotype contrasts, the significance level (p value) of heterogeneity test (Q test), the I^2^ metric and publication bias p-value (Egger Test) in total studies, Asian, American and European studies.

GeneticContrast		Fixed effect OR(95% CI), p	Random effect OR(95% CI), p	Heterogeneityp-value (Q test)	I^2^ (%)	Publication Bias(p of Egger’s test)
All	Allele Contrast (T vs. C)	1.22 (1.13−1.31), <0.0001	1.26 (1.09−1.46), 0.001	<0.0001	69.42	0.14
	Co-dominant (CT vs. CC)	1.23 (1.11−1.36), <0.0001	1.29 (1.10−1.51), 0.001	0.0002	52.49	0.02
	Homozygote (TT vs. CC)	1.44 (1.22−1.69), <0.0001	1.49 (1.13−1.97), 0.008	<0.0001	57.3	0.56
	Dominant (TT+CT vs. CC)	1.28 (1.16−1.41), <0.0001	1.35 (1.13−1.60), 0.0008	<0.0001	63.56	0.05
	Recessive (CT+CC vs. TT)	0.76 (0.65−0.88), 0.0004	0.76 (0.60−0.94), 0.01	0.0044	43.68	0.926
Asian	Allele Contrast (T vs. C)	1.53 (1.29−1.82), <0.0001	1.52 (1.09−2.1), 0.01	0.0003	69.43	0.82
	Co-dominant (CT vs. CC)	1.52 (1.21−1.91), 0.0003	1.57 (1.14−2.14), 0.005	0.09	38.05	0.11
	Homozygote (TT vs. CC)	2.41 (1.62−3.59), <0.0001	2.21 (1.03−4.74), 0.0411	0.0074	60.04	0.204
	Dominant (TT+CT vs. CC)	1.64 (1.32−2.0), <0.0001	1.70 (1.18−2.4), 0.004	0.01	56.67	0.30
	Recessive (CT+CC vs. TT)	0.54 (0.37−0.78), <0.0001	0.58 (0.29−1.16), 0.12	0.0094	58.77	0.334
American	Allele Contrast (T vs. C)	1.23 (1.07−1.39), 0.003	1.19 (0.99−1.44), 0.06	0.06	47.69	0.11
	Co-dominant (CT vs. CC)	1.42 (1.17−1.71), 0.0002	1.42 (0.97−2.06), 0.066	0.0005	73.15	0.908
	Homozygote (TT vs. CC)	1.68 (1.24−2.28), 0.0008	1.58 (0.84−2.95), 0.148	0.0007	72.07	0.667
	Dominant (TT+CT vs. CC)	1.48 (1.24−1.76), <0.0001	1.44 (0.95−2.19), 0.078	<0.0001	80.11	0.782
	Recessive (CT+CC vs. TT)	0.69 (0.51−0.92), 0.0136	0.72 (0.44−1.18), 0.203	0.0159	59.42	0.753
European	Allele Contrast (T vs. C)	1.03 (0.93−1.15), 0.482	1.04 (0.93−1.16), 0.451	0.3576	8.81	0.084
	Co-dominant (CT vs. CC)	0.99 (0.85−1.16), 0.956	1.00 (0.85−1.17), 0.992	0.3774	6.87	0.050
	Homozygote (TT vs. CC)	1.09 (0.87−1.37), 0.422	1.09 (0.85−1.40), 0.455	0.3715	7.45	0.329
	Dominant (TT+CT vs. CC)	1.02 (0.88−1.17), 0.787	1.03 (0.87−1.21), 0.704	0.308	13.58	0.041
	Recessive (CT+CC vs. TT)	0.90 (0.73−1.10), 0.322	0.90 (0.72−1.11), 0.339	0.570	0	0.948

**Figure 3:**
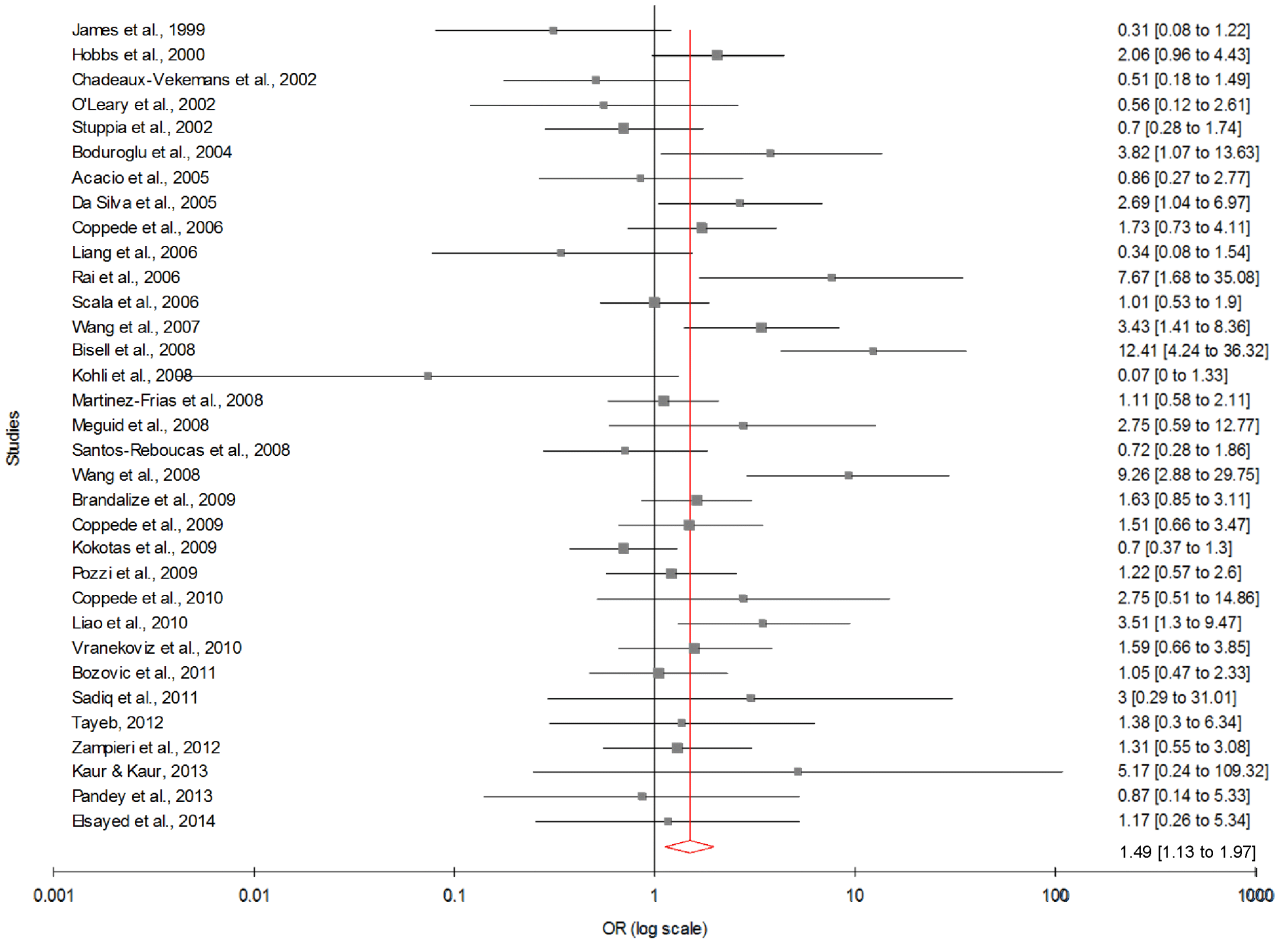
Forest plots (Random effect) showed significant association between MTHFR C677T polymorphism and risk of Down syndrome. Results of individual and summary OR estimates and 95% CI of each study were shown using homozygote model (TT versus CC).

**Figure 4:**
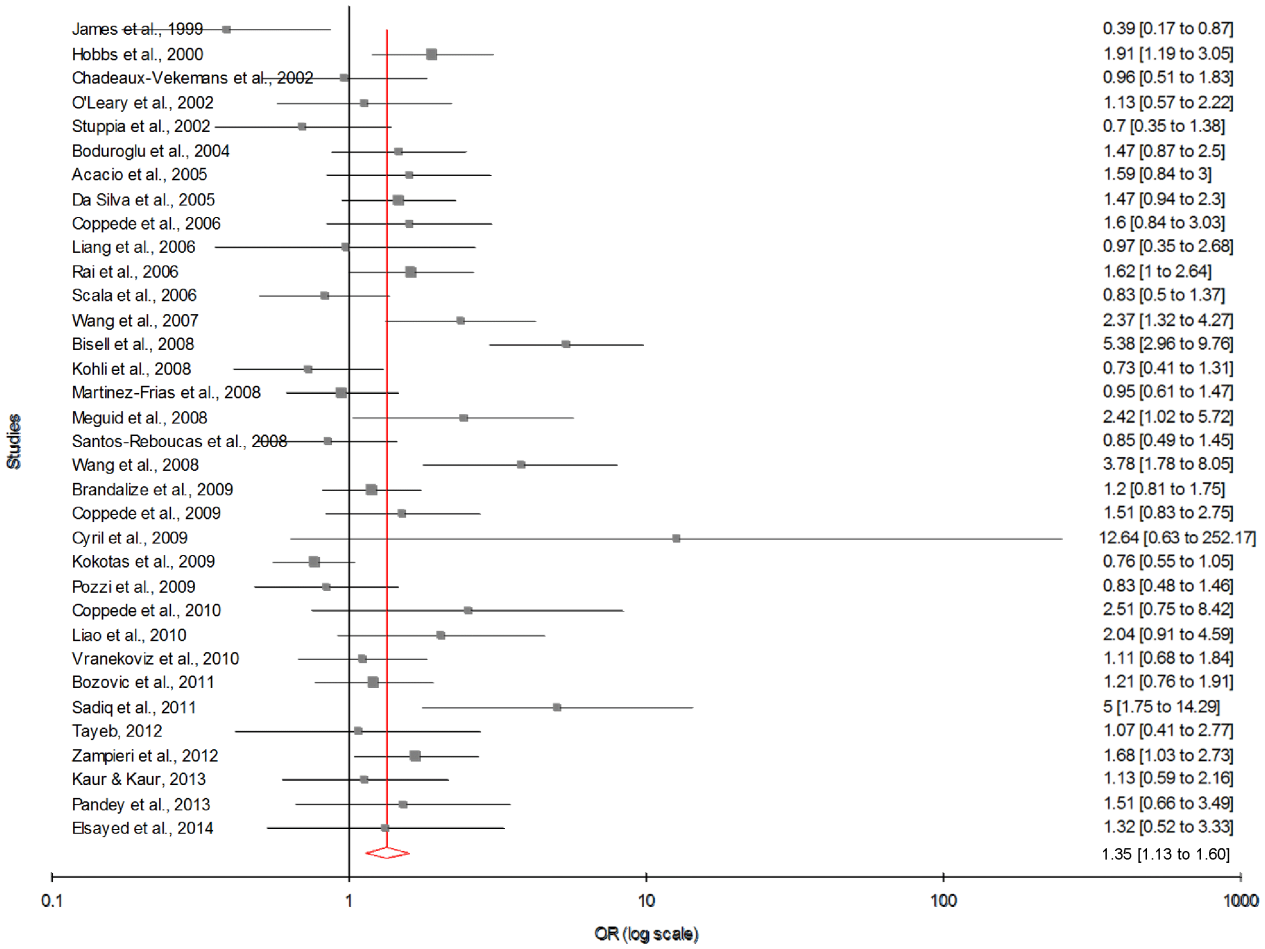
Forest plots (Random effect) showed significant association between MTHFR C677T polymorphism and risk of Down syndrome using dominant model (TT+CT versus CC). Results of individual and summary OR estimates and 95% CI of each study were shown.

Table 3 summarizes the ORs with corresponding 95% CIs for association between maternal C677T polymorphism and risk of DS in dominant, recessive, homozygote and co-dominant models. With our primary analysis, there was an increased risk of DS among mutant homozygote variants (TT), with both fixed (OR_TTvs.CC_ = 1.44; 95% CI = 1.22−1.69, p = <0.0001) and random (OR_TTvs.CC_ = 1.49; 95% CI = 1.13−1.97, p = 0.008) effect models with moderate statistical heterogeneity between-study (Figure 3). Association of mutant heterozygous genotype (CT vs. CC) was observed significant with fixed (OR_CTvs.CC_ = 1.23; 95% CI = 1.11−1.36; p = <0.0001) and random (OR_CTvs.CC_ = 1.29; 95% CI = 1.10−1.51; p = 0.001) effect models. Similarly combined mutant genotypes (TT+CT vs. CC) showed significant association with DS using both fixed (OR_TT+CTvs.CC_ = 1.28; 95% CI = 1.16−1.41; p = <0.0001) and random (OR_TT+CTvs.CC_ = 1.35; 95% CI = 1.13−1.60; p = 0.0008) effect models (Figure 4).

### Stratified analysis

**Figure 5:**
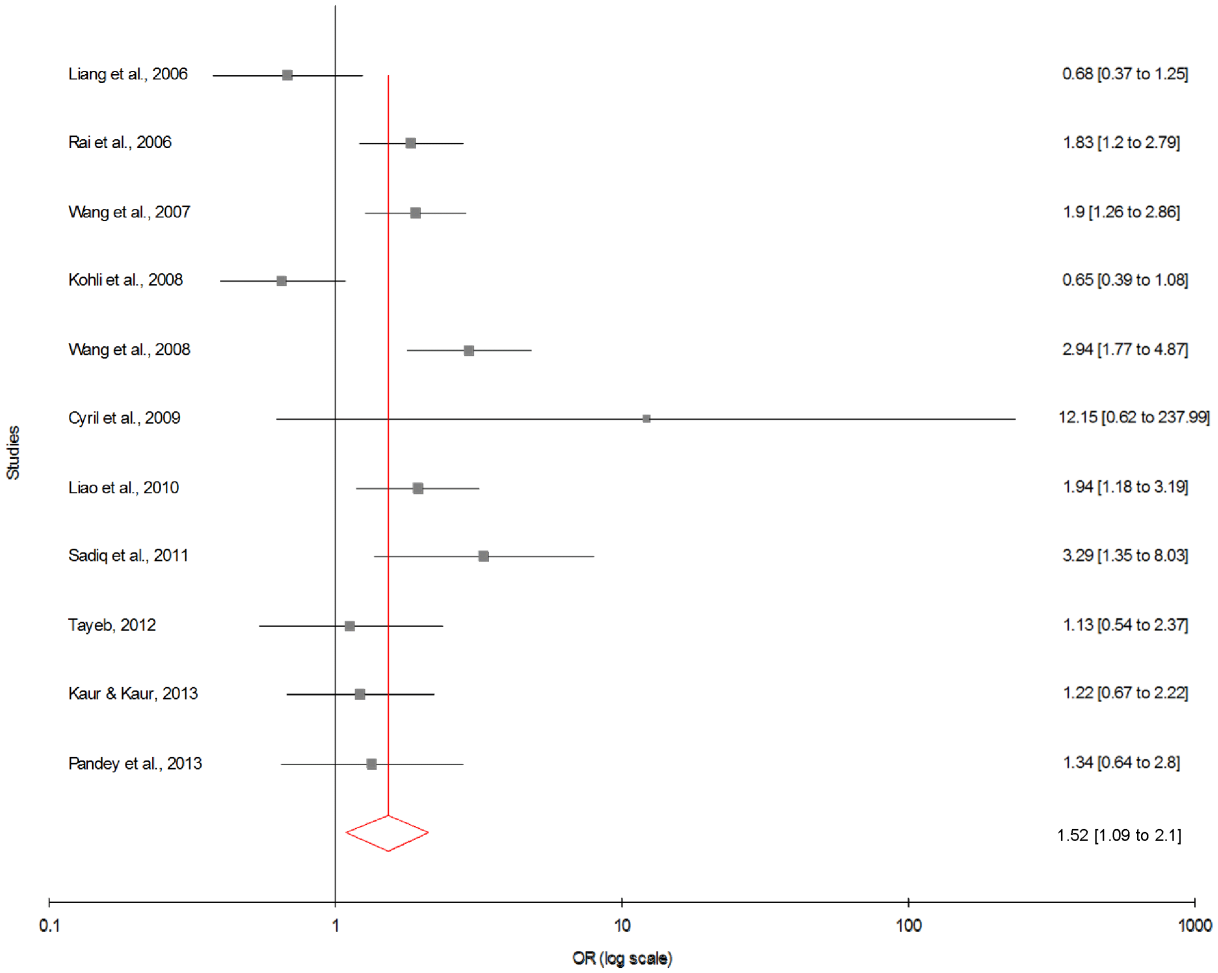
Forest plots (Random effect) showed significant association between MTHFR C677T polymorphism and risk of Down syndrome in Asian studies using allele contrast model (T versus C). Results of individual and summary OR estimates and 95% CI of each study were shown.

**Figure 6:**
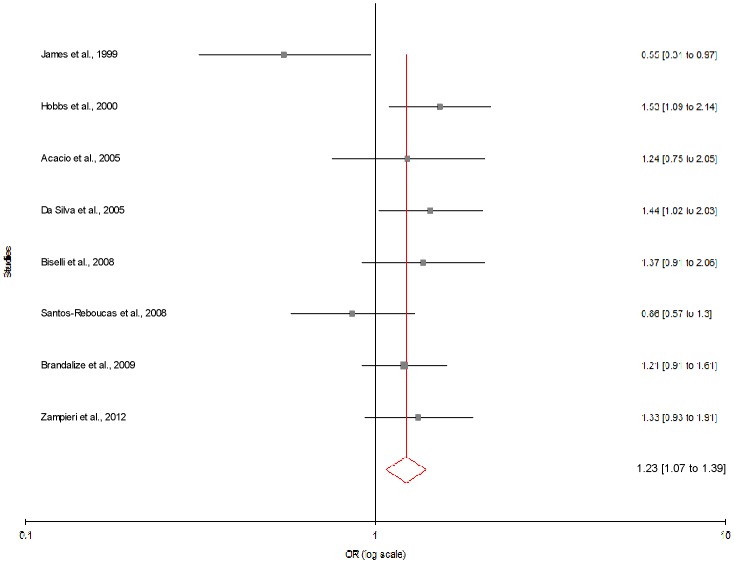
Forest plots (Random effect) showed no association between MTHFR C677T polymorphism and risk of Down syndrome in American studies using allele contrast model (T versus C). Results of individual and summary OR estimates and 95% CI of each study were shown.

**Figure 7:**
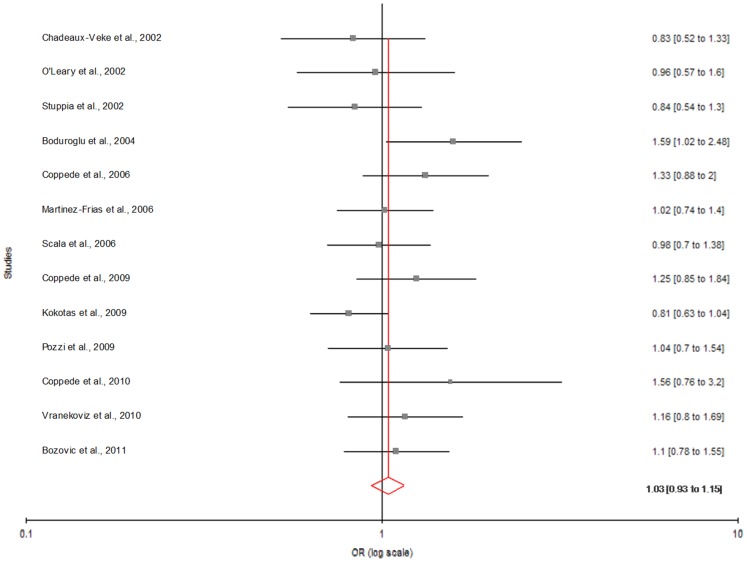
Forest plots (Fixed effect) showed no association between MTHFR C677T polymorphism and risk of Down syndrome in European studies using allele contrast model (T versus C). Results of individual and summary OR estimates and 95% CI of each study were shown. Horizontal lines represented 95% CI, and dotted vertical lines represent the value of the summary OR.

We also performed sub-group analysis which is based on geographic distribution of population. Out of 34 studies included in present meta-analysis, 11 studies were from Asia, 13 from Europe, 8 from America and 2 from Africa. The subgroup analysis by geographical regions revealed that the significant association between the maternal *MTHFR* C677T polymorphism and DS existed in Asian population (for T vs. C: OR = 1.51; 95% CI = 1.09−2.10; p = 0.01; I^2^ = 69.43%; P_heterogeneity_ = 0.0003; P_Pb_ = 0.82) (Figure 5; Table 3). Except allele contrast model of American population (T vs. C: OR = 1.23; 95% CI = 1.07−1.39; p = 0.003; I^2^ = 47.69%; P_heterogeneity_ = 0.06; P_Pb_ = 0.11) (Figure 6) no significant association was found in American and European population (for T vs. C: OR = 1.03; 95% CI = 0.93−1.15; p = 0.482; I^2^ = 8.81%; P_heterogeneity_ = 0.357; P_Pb_ = 0.084) (Figures 7; Table 3).

### Heterogeneity and Sensitive analysis

A true heterogeneity existed between studies for allele (P_heterogeneity_ = <0.0001, Q = 107.92, df = 33, I^2^ = 69.42%, t^2^ = 0.12) and mutant genotypes (P_heterogeneity_ = <0.0001, Q = 74.90, df = 32, I^2^ = 57.3%, t^2^ = 0.10) comparisons. The ‘I^2^’ value of more than 50% for between studies comparison in both allele and genotype analysis shows high level of true heterogeneity. In Asian (P_heterogeneity_ = 0.0003, I^2^ = 67.43%) and American (P_heterogeneity_ = <0.0001, I^2^ = 83.25%) allele contrast meta-analysis significant high heterogeneity was observed, in European sub-group meta-analysis low heterogeneity was observed (P_heterogeneity_ = 0.357, I^2^ = 8.81) in allele contrast model.

In allele contrast meta-analysis, sensitivity analysis performed by exclusion of the studies in which control population was not in Hardy Weinberg equilibrium, studies with small sample size and studies with high p values. Control population of only two studies [28], [43] were not in HW equilibrium and heterogeneity did not decreased after exclusion of these studies (p = <0.0001, I^2^ = 70.00%). Exclusion of seven studies with small sample size, less than 50 (O’Leary et al. [44], n = 41; Liang et al. [34], n = 30; Mequid et al [38], n = 42; Cyril et al. [42], n = 36; Coppede et al. [48], n = 29; Tayeb [2], n = 30; Elsayed et al. [39], n = 26), also did not decreased heterogeneity (P_heterogeneity_ = <0.0001, I^2^ = 72.98%). Similarly exclusion of eleven studies with very high p value (O’Leary et al. [44], p = 0.87; Acacio et al. [28], p = 0.40; Scala et al. [7], p = 0.91; Martinez-Frias et al. [51], p = 0.90; Pozzi et al. [13], p = 0.84;Vranekoviz et al. [37], p = 0.43; Bozovic et al. [8], p = 0.58; Tayeb [2], p = 0.74; Elsayed et al. [39], p = 0.65; Kaur and Kaur [5], p = 0.52; Pandey et al. [43], p = 0.44) did not decrease heterogeneity but increased odds ratio (OR = 1.29, 95% CI = 1.18−1.41, p = <0.0001).

### Publication bias

**Figure 8:**
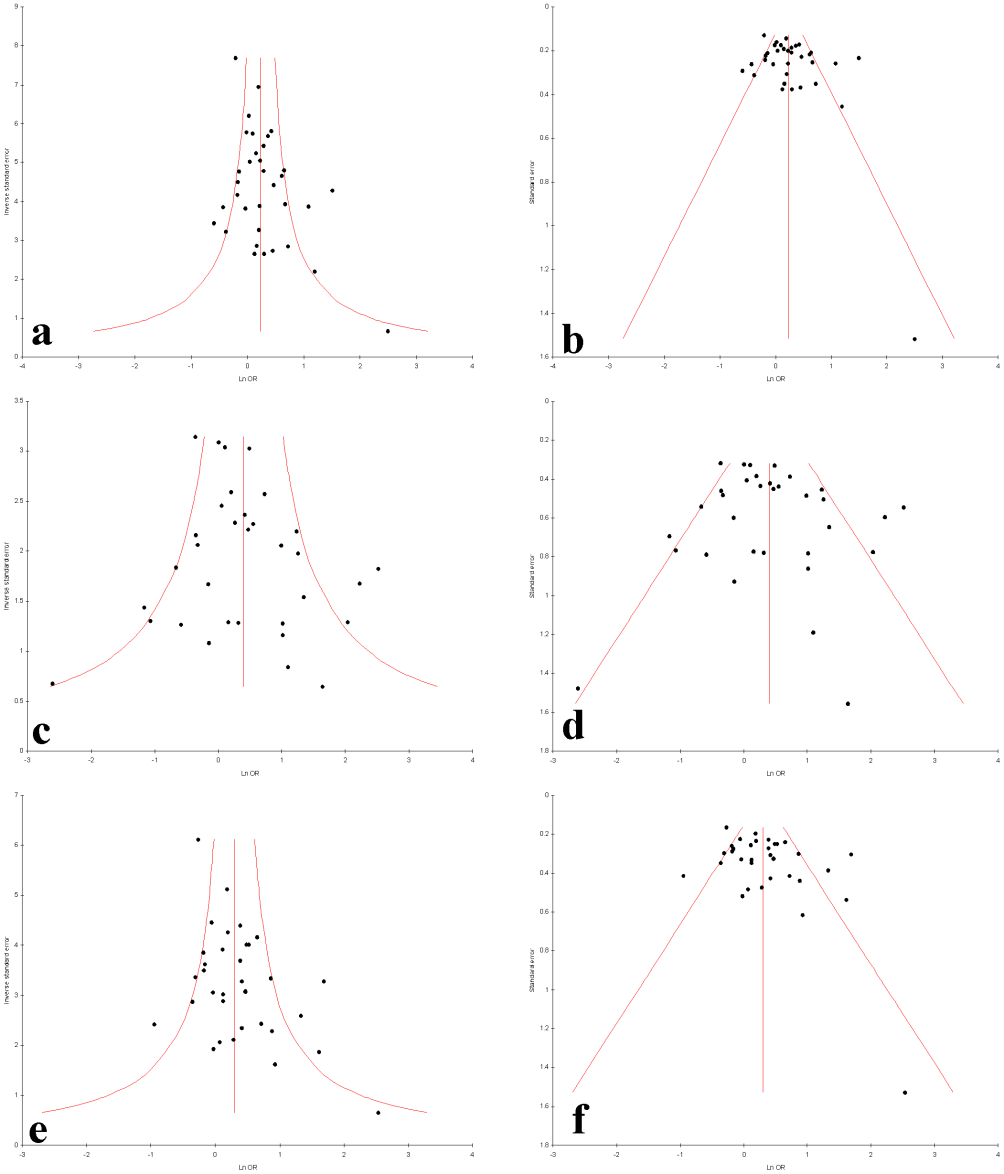
Funnel plots a−f. a. Precision by log odds ratio for additive model; **b.** standard error by log odds ratio for additive model; **c.** precision by log odds ratio for co-dominant model; **d.** standard error by log odds ratio for co-dominant model; **e.** precision by log odds ratio for dominant model; **f.** standard error by log odds ratio for Dominant model.

**Figure 9:**
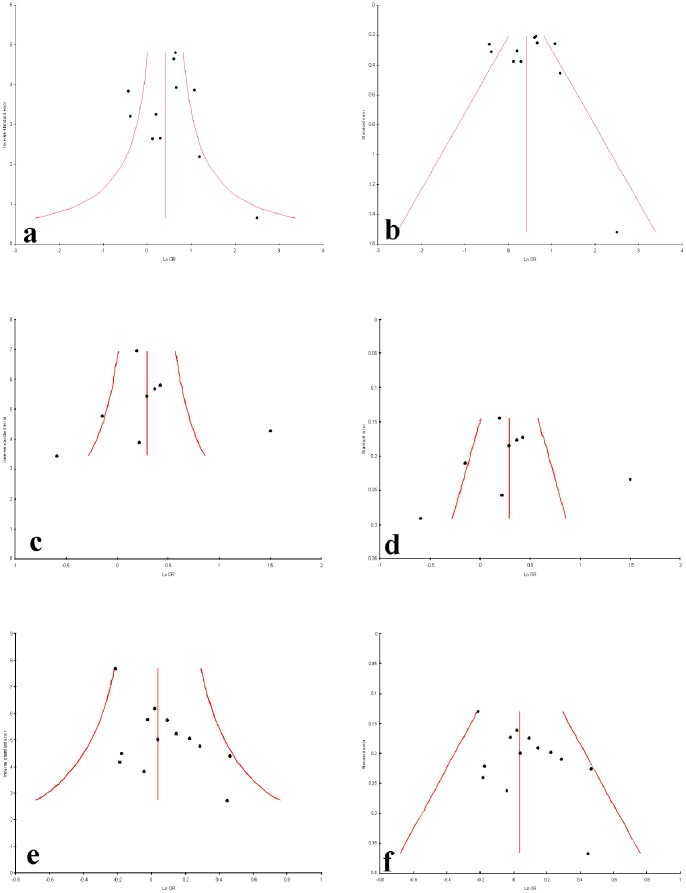
Funnel plots a−f. a. Precision by log odds ratio for additive model; **b.** standard error by log odds ratio for additive model for Asian studies; **c.** precision by log odds ratio for additive model; **d.** standard error by log odds ratio for additive model for American studies; **e.** precision by log odds ratio for additive model; **f.** standard error by log odds ratio for additive model for European studies.

Publication bias was not observed in allele contrast, homozygote, dominant and recessive models (Begg’s p = 0.28, Egger’s p = 0.14 for T vs. C; Begg’s p = 0.38, Egger’s p = 0.56 for TT vs. CC; Begg’s p = 0.13, Egger’s p = 0.05 for TT+CT vs. CC and Begg’s p = 0.19, Egger’s p = 0.0.05 for TT vs. CC+CT) but publication bias was observed in co-dominant model (Begg’s p = 0.04, Egger’s p = 0.02 for CT vs. CC) of overall by using Begg’s and Egger’s test (Table 3). Funnel plots were showed in Figures 8 and 9.

## Discussion

In 1999, James et al [3] reported that genetic polymorphism of folate and homocysteine pathway enzymes predispose a woman to abnormal chromosome segregation, which act as risk factor for DS pregnancy. In subsequent years, several in vivo studies in humans suggested that chronic folate deficiency has been associated with abnormal DNA methylation [11], [53], [54], and aberrant chromosome segregation [6], . Population-based studies have shown that folic acid intake during fetal development has a protective effect, resulting in a significant reduction in the occurrence of developmental defects, like neural tube defects (NTD), congenital heart defects, limb defects, and orofacial clefts [60].

Meta-analysis is a powerful tool for analyzing cumulative data with small and low power studies. Several meta-analyses were published accessing *MTHFR* as risk factor to various diseases/disorders like- neural tube defects [61], [62], cleft lip and palate [63], stroke [64], psychiatric disorders [65]. During literature search, we identified four meta-analyses [66]–[69] published between 2007 and 2013. They examined the effect of maternal *MTHFR* C677T as DS risk, but no consistent conclusion was achieved. Zintzaras [66] performed a meta-analysis based on eleven studies and did not find any significant association between the maternal *MTHFR* polymorphisms and DS risk. Medica et al. [67] aggregated sixteen studies and reported significant relationship between the maternal mutant genotypes (TT+CT vs CC) and risk of DS child. Recently, Wu et al. [68] published a meta-analysis (included twenty eight studies with 2806 cases/4597 controls), and found statistical association with dominant model (OR = 1.305, 95% CI = 1.125–1.514, p = 0, p = 0.003). Yang et al. [69] performed a meta-analysis which was based on twenty six studies (2458 cases/3144 controls) and found statistically significant association in allele contrast model (OR = 1.28; 95% CI: 1.11–1.47) (Table 4). Several newly published studies were not included in the previous published meta-analyses. So authors conducted a comprehensive meta-analysis with the largest number of studies (34 studies). In the present meta-analysis significant association was found between maternal C677T polymorphism and DS risk in total 34 studies using all five genetic models. Whereas in stratified analysis, except allele contrast model in American population, no significant association was observed in European and American population but significant higher risk was found in Asian population. Such phenomenon probably could be ascribed to the folate metabolism profile and dietary structure of different regions.

**Table 4:** A comparative analysis of details of Odds Ratio, 95% CI, genetic models reported in total 5 (including present) meta-analysis published so far analyzing case-control studies of *MTHFR* C677T polymorphism and Down syndrome.

Study	Number of Studies	Cases	Controls	I^2^ (%)	Heterogeneity p-value (Q test)	OR (95% CI), p-value	Model	Subgroup analysis
Zintaras, 2007	11	1129	1489	49	0.03	1.20 (1.06–1.35)	Allelic contrast	Not reported
Medica et al., 2009	16	1545	2052	–	–	1.40 (1.16–1.70), 0.0006	Dominant model	Not reported
Yang et al., 2013	26	2458	3144	58.2	<0.01	1.28 (1.11–1.47)	Allelic contrast	Reported
Wu et al., 2013	28	2806	4597	48.0	0.0	1.224 (1.085–1.38), 0.001	Dominant model	Reported
Present Study, 2014	34	3048	4852	69.42	<0.0001	1.26 (1.09–1.46), 0.001	Allelic contrast	Reported

There are few limitations of the present meta-analysis like- i) we used crude ORs in the pooled analysis without adjustment; ii) the relatively small sample size in some of the included studies, especially those from Asia; iii) we considered only one gene polymorphism (*MTHFR* C677T) of folate pathway. Present meta-analysis had several advantages/strength to the previous published meta-analyses like- (i) the publication bias was not detected in present meta-analysis, (ii) pooled number of cases and controls from different studies significantly increased the statistical power of the analysis, (iii) largest number of studies (34 studies) with largest sample size (3,098 cases and 4,852 controls) was included in the present meta-analysis, (iv) controls included in the present meta-analysis was mothers of healthy child, (v) distribution of genotypes in control mothers except two studies was in Hardy-Weinberg equilibrium, (vi) significant association was found between maternal *MTHFR* C677T polymorphism and DS risk in allelic contrast, homozygote, co-dominant and dominant genetic models and (vii) in addition we did sub-group analysis according to geographical regions.

In conclusion, results of present meta-analysis suggest that the maternal *MTHFR* 677T allele is a risk factor for development of DS pregnancy. However the results of present meta-analysis were based on single gene polymorphism and significant heterogeneity was also observed; hence results should be interpreted with caution.

## Supporting Information

Checklist S1 **PRISMA checklist.**
(DOC)
